# Starvation and Inflammation Modulate Adipose Mesenchymal Stromal Cells’ Molecular Signature

**DOI:** 10.3390/jpm14080847

**Published:** 2024-08-09

**Authors:** Simona Piccolo, Giulio Grieco, Caterina Visconte, Paola De Luca, Michela Taiana, Luigi Zagra, Enrico Ragni, Laura de Girolamo

**Affiliations:** 1 Laboratorio di Biotecnologie Applicate all’Ortopedia, IRCCS Istituto Ortopedico Galeazzi, Via Cristina Belgioioso 173, 20157 Milano, Italy; simona.piccolo@grupposandonato.it (S.P.); giulio.grieco@grupposandonato.it (G.G.); caterina.visconte@grupposandonato.it (C.V.); deluca.paola@grupposandonato.it (P.D.L.); michelamaria.taiana@grupposandonato.it (M.T.); laura.degirolamo@grupposandonato.it (L.d.G.); 2 Hip Department, IRCCS Istituto Ortopedico Galeazzi, Via Cristina Belgioioso 173, 20157 Milano, Italy; luigizagra@grupposandonato.it

**Keywords:** adipose mesenchymal stromal cells, secretome, starvation, inflammation, osteoarthritis, chondrocytes

## Abstract

Mesenchymal stromal cells (MSCs) and their released factors (secretome) are intriguing options for regenerative medicine approaches based on the management of inflammation and tissue restoration, as in joint disorders like osteoarthritis (OA). Production strategy may modulate cells and secretome fingerprints, and for the latter, the effect of serum removal by starvation used in clinical-grade protocols has been underestimated. In this work, the effect of starvation on the molecular profile of interleukin 1 beta (IL1β)-primed adipose-derived MSCs (ASCs) was tested by assessing the expression level of 84 genes related to secreted factors and 84 genes involved in defining stemness potential. After validation at the protein level, the effect of starvation modulation in the secretomes was tested in a model of OA chondrocytes. IL1β priming in vitro led to an increase in inflammatory mediators’ release and reduced anti-inflammatory potential on chondrocytes, features reversed by subsequent starvation. Therefore, when applying serum removal-based clinical-grade protocols for ASCs’ secretome production, the effects of starvation must be carefully considered and investigated.

## 1. Introduction

Mesenchymal stromal cells (MSCs) have emerged as a promising option for therapeutic applications where the management of inflammation and the restoration of tissue homeostasis are required [1]. Considering the release of different messengers, collectively defined as the “secretome”, the paracrine pathway is implicated in the therapeutic properties of MSCs [2]. Also, the influence of the local microenvironment, especially immunological and inflammatory signals, may modulate the mechanisms underlying the therapeutic effects [3]. For these reasons, several priming strategies aimed at improving MSCs’ potential were developed [4], including inflammatory molecules, hypoxia, or 3D cultures. Under these premises, MSCs were tested in several preclinical models, with primed cells showing improved efficacy [4]. Those results led to the implementation of more than 1500 clinical trials to treat various pathologies, according to the US National Institute of Health Clinical Trials database (http://clinicaltrials.gov (accessed on 15 April 2024)). By topic, musculoskeletal diseases represented the most studied condition (22% of total studies), with the main focus on osteoarthritis (OA) (12%). The common feature of preclinical and clinical studies is the preparation of cells under good laboratory (GLP, preclinical) or good manufacturing practice (GMP, clinical), often their storage, and finally their administration [5]. Therefore, cells, even when primed, are delivered to patients after their expansion, and their response is the combination of the conditions during their production followed by those cells’ encounters depending on the pathology to be treated.

Recently, due to MSCs’ activity mainly relying on paracrine factors, the therapeutic application of MSCs’ secretome is under study as a safer option than cell transplantation since it reduces the concern for tumorigenesis and immunogenicity [6]. Considering the path paved by these cells, at present, more than 20 clinical trials are registered in the US National Institute of Health Clinical Trials database, with 21% related to musculoskeletal diseases and 12% to OA. The first completed study evaluated the effect of the secretome from umbilical MSCs in patients with mild-to-moderate symptomatic knee OA (NCT05579665). The results showed that secretome intra-articular injections led to superior clinical improvement, biomarker changes, and no side effects compared to hyaluronic acid over a 5-week interval [7]. The major drawback of secretomes is that, differently from cells, they cannot adapt to environmental cues, and therefore their potential is strictly related to their composition and the effects of the manufacturing process under critical analysis. In this regard, similarly to the medium supplements used during GLP/GMP expansion, such as FBS or human platelet lysate/serum, which have to be “washed out” from cells before their administration to patients, secretomes must also be deprived of these components. Waiting for new serum/xeno-free chemically defined media [8], the most used strategy for secretome production in view of clinical translation is starvation after cell expansion [9]. The issue of serum removal is underestimated since the results obtained with secretomes produced in the presence of serum are often considered applicable for starved secretomes, disregarding the effects of supplement deprivation on cells and their released factors. As an example, in ASCs, serum starvation affects mitochondrial metabolism [10], which is directly responsible for their secretory profile [2]. Consistently, in one of the rare comparative studies, secretomes collected under serum conditions did not have the same extent of positive effects as those collected under starvation for pulp repair [11]. Under these premises, if MSCs can be primed before their collection and delivered to patients to improve their efficacy, a similar approach before secretome collection might be debatable since starvation could affect the secretory portfolio of primed cells.

The main goal of this work was to study the molecular fingerprint of ASCs cultivated in the presence or absence of inflammatory priming and evaluate the effect of subsequent starvation. Gene expression results were validated at the protein level, and the different secretomes were tested on an in vitro model of inflamed chondrocytes.

## 2. Materials and Methods

### 2.1. Materials and Methods

#### 2.1.1. Ethics

This study was performed under IRB approval (San Raffaele Hospital Ethics Committee, 16 December 2020, N° 214/int/2020) and followed the 1964 Helsinki Declaration and its later amendments or comparable ethical standards. Informed consent was obtained from patients.

#### 2.1.2. ASC Isolation and Culture

Adipose tissue was collected from three female donors (median 32 y/o ± 3) undergoing elective plastic surgery and digested for 30 min at 37 °C with 0.075% w/v type I collagenase (Worthington Biochemical Co, Lakewood, NJ, USA). After 100 µm cell strainer filtering, the cells were seeded at 5 × 10^3^ cells/cm^2^ in DMEM + 10% FBS (Thermofisher Scientific, Waltham, MA, USA) and cultured at 37 °C with 5% CO_2_ and 95% humidity. The full characterization of ASCs isolated with this procedure was reported in Stanco et al. [12]. Experiments were performed using passage 2 on individual donors, who were kept separate. Five conditions were set up as follows:

(i) “F”: 48 h in a complete medium;
(ii) “F+IL”: as “F” supplemented with 1 ng/mL interleukin 1 beta (IL1β);
(iii) “S”: 48 h without serum (starvation), after 48 h in a complete medium;
(iv) “S+IL”: as “S” supplemented with 1 ng/mL IL1β;
(v) “F+IL/S”: 48 h without serum (starvation), after 48 h in a complete medium supplemented with 1 ng/mL IL1β.

#### 2.1.3. Flow Cytometry

The following antibodies were used: anti-CD34-PE, CD73-PE, CD90-FITC, CD45-PE Vio770 (Miltenyi Biotec, Bergisch Gladbach, Germany), and CD44-PerCP (Immunostep, Salamanca, Spain). A minimum of 30,000 events were acquired with a CytoFLEX flow cytometer (Beckman Coulter, Fullerton, CA, USA). Isotype controls did not show specific staining. CytExpert v2.3 (Beckman Coulter) software was used to process data.

#### 2.1.4. Gene Expression Analysis with PCR Arrays

Cells were collected in a QIAzol reagent (Qiagen, Hilden, Germany). RNA was purified using the miRNeasy Kit (Qiagen). Briefly, 1.6 µg of each purified RNA was retrotranscribed using the RT^2^ First Strand Kit (Qiagen), and the reaction mix was dispensed in RT^2^ Profiler PCR Array 384 format (Qiagen; PAHS-150ZA and PAHS-082ZA). PCR reactions were performed with the QuantStudio™ Real-Time PCR system (Thermofisher Scientific). Data were analyzed using web-based software (Qiagen) after normalization on *HPRT1* [13].

#### 2.1.5. ELISA Assays

Secretomes were analyzed with the following ELISA kits: IL-8, G-CSF, GM-CSF (900-M30), CCL2, IL-6, and CXCL2 (Peprotech, London, UK). Samples were used without dilution or after a dilution of 1:10/1:100 to ensure the absorbance readings fell within the standard curve. Absorbance was measured on Microplate Reader Victor^TM^ (PerkinElmer, Waltham, USA) and compared with standard samples. Similarly, IL1β (Peprotech, London, UK) was tested to evaluate its stability and residual amount in secretomes before its addition to inflamed chondrocytes for functional experiments.

#### 2.1.6. Functional Experiment on Chondrocytes

Immortalized chondrocytes (InSCREENex GmbH, Braunschweig, Germany) were seeded at 90,000 cells/cm^2^ in a complete medium. The next day, the medium was discarded, and the wells were washed with PBS. Control wells were cultured with a complete medium, while the other wells were cultured with 1 ng/mL IL1β added to either a complete medium or a pool of secretomes for each different condition (F, F+IL, S, S+IL, and F+IL/S). All wells were adjusted to a 20% FBS final concentration. Secretomes were used undiluted. After 48 h, cells were collected with QIAzol. RNA was extracted with the miRNeasy Micro Kit, and RNA was retrotranscribed using the iScriptTM cDNA Synthesis Kit (BioRad, Hercules, CA, USA). The amplifications were conducted in a CFX Opus Real-Time PCR System (BioRad). The following genes were assayed: *IL1B*, *IL6*, *IL8*, *CTSS*, *IDO*, and *CCL2*. To determine the fold change, the 2^−ΔΔCt^ method was employed after *TBP* normalization.

#### 2.1.7. Hierarchical Clustering

The ClustVis package (https://biit.cs.ut.ee/clustvis/, accessed on 19 March 2024) was used to perform principal component analysis (PCA) and hierarchical clustering. Maps were generated using the following settings for both rows’ and columns’ clustering method/distance: average and correlation, respectively. Untransformed values or ln(X + 1) values were used.

#### 2.1.8. Statistical Analyses

The Shapiro–Wilk normality test (α of 0.01) was performed to test for normal data distribution. With normal values, a one-way RM-ANOVA was performed; otherwise, a Friedman test was conducted with the exception of mean gene modulation in chondrocytes where a one-sample *t*-test for each condition vs. IL1β set as 1 was performed (GraphPad Prism v8.0.2; GraphPad Software, San Diego, CA, USA).

## 3. Results

### 3.1. Results

#### 3.1.1. ASCs’ Immunophenotype

**Figure 1:**
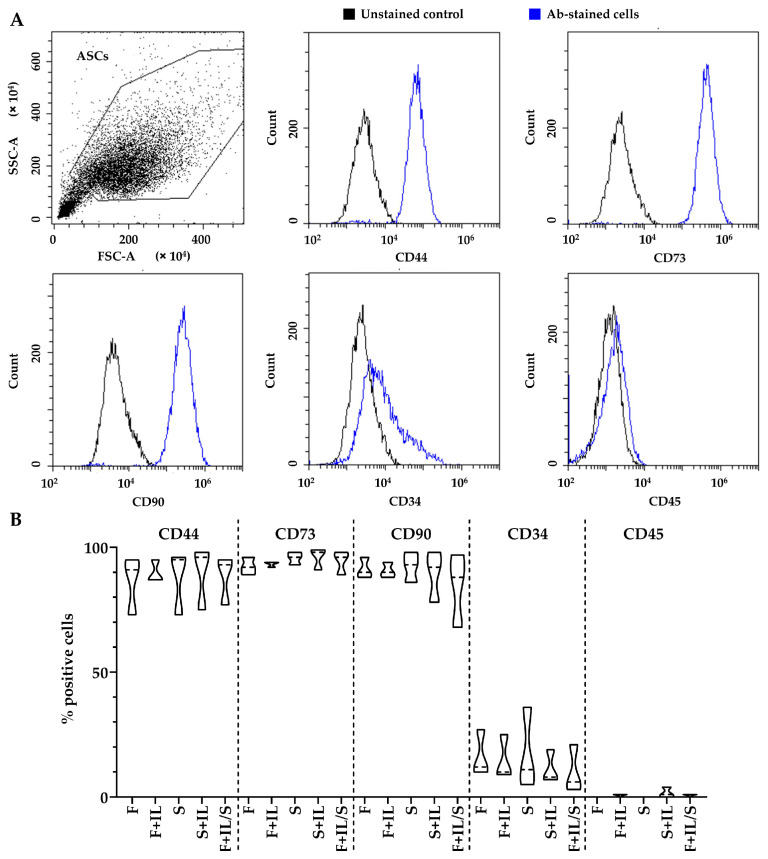
ASCs’ immunophenotype: (**A**) ASCs were positive for MSC markers CD44, CD73, CD90, and CD105, and tissue-resident/early-passage ASC marker CD34. Plots illustrate the results from a representative donor. (**B**) No difference was observed under the analyzed conditions (N = 3).

ASCs were positive for MSC markers CD44, CD73, and CD90 (Figure 1A). The early ASC marker CD34 [14] was also detectable (Figure 1A). No differences emerged under the different tested conditions (Figure 1B).

#### 3.1.2. Inflammation and Starvation Affect ASCs’ Molecular Signature Related to Inflammation Markers

**Figure 2:**
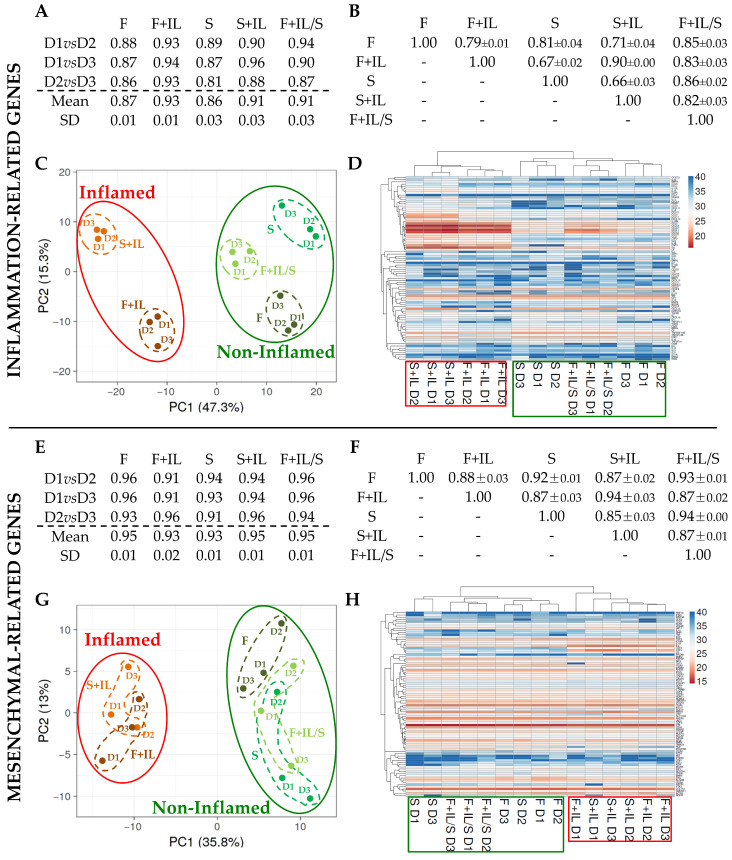
Correlation between samples and conditions: (**A**) Intra-group and (**B**) inter-group correlation analyses for the expression of inflammation-related genes; N = 3, mean ± SD. “*r*” Pearson values are shown. (**C**) PCA performed on normalized C_t_ values for inflammation genes. The X and Y axes show principal component 1 and principal component 2, which explain 47.3% and 15.3% of the total variance. (**D**) Hierarchical clustering performed on normalized C_t_ values for inflammation genes. Higher Ct means lower amount, and lower Ct means higher amount. (**E**) Intra-group and (**F**) inter-group correlation analyses for the expression of mesenchymal stem cell-related genes; N = 3, mean ± SD. “*r*” Pearson values are shown. (**G**) PCA performed on normalized Ct values for mesenchymal genes. The X and Y axes show principal component 1 and principal component 2, which explain 35.8% and 13.0% of the total variance. (**H**) Hierarchical clustering performed on normalized Ct values for mesenchymal genes. Higher Ct means lower amount, and lower Ct means higher amount.

ASCs were first tested for the expression of 84 genes coding for inflammation-related cytokines, chemokines, and growth factors. Intra-group correlation analysis showed that the three donors behaved similarly, with high “*r*” values (Figure 2A), namely 0.87 ± 0.01 for F, 0.93 ± 0.01 for F+IL, 0.86 ± 0.03 for S, 0.91 ± 0.03 for S+IL, and 0.91 ± 0.03 for F+IL/S. Inflammatory priming, regardless of the presence or absence of serum, promoted a higher homogeneity between donors that lasted after starvation. This was confirmed by statistical analysis, which showed a significant difference in intra-group F+IL correlation value vs. intra-group F value (*p*-value of 0.0439), and a similar trend was observed taking into account S+IL vs. S (*p*-value of 0.0976). Additionally, considering inter-group correlation scores, the presence of inflammation was able to separate F+IL and S+IL samples (Figure 2B). The lowest *r* values were obtained for F+IL vs. F (0.079 ± 0.01), F+IL vs. S (0.67 ± 0.02), S+IL vs. S (0.66 ± 0.03), and S+IL vs. F (0.71 ± 0.04). Accordingly, the highest *r* value was for the samples under inflammation, i.e., F+IL vs. S+IL (0.90 ± 0.00). This result confirmed again the homogeneity prompted by IL1β since F vs. S had a lower score of 0.81 ± 0.04. PCA confirmed the dichotomy between conditions with inflamed samples, which were again separated from those untreated, with S+IL having the most divergent position (Figure 2C). This result was substantiated by hierarchical clustering (Figure 2D). Notably, similar to PCA results, samples starved after inflammation (F+IL/S) clustered together with F and S.

#### 3.1.3. Inflammation and Starvation Affect ASCs’ Molecular Signature Related to Mesenchymal Markers

For this analyzed gene set, the high intra-group correlation between donors was confirmed with *r* values always higher than 0.9 (Figure 2E). Nevertheless, as shown for soluble factors, inflammation resulted in a dichotomy between samples (Figure 2F). Inter-group correlation values were lower for samples not inflamed than for samples collected under IL1β treatment with respect to the values obtained for the comparisons of non-inflamed or inflamed samples. In fact, the *r* values for F vs. F+IL and F vs. S+IL or S vs. S+IL and S vs. F+IL, as well as F+IL/S vs. F+IL or S+IL, were always <0.9. This result was corroborated by the high homogeneity of samples under inflammation, with F+IL vs. S+IL having the highest *r* (0.94 ± 0.03), which was also observed for F+IL/S vs. S, with an even lower standard deviation (0.94 ± 0.00). These data were confirmed by PCA (Figure 2G) and hierarchical clustering (Figure 2H), in which inflamed and non-inflamed samples were sharply separated. Interestingly, in the PCA plot, S+IL and F+IL data points, as well as S and F+IL/S, were almost superimposed. In addition, the more fine-tuned analysis given by hierarchical clustering encompassing the gene expression patterns again enabled the separation of the different conditions, with the exception of donor D2 in the S condition falling in the F group and donor D1 in F+IL closer to S+IL ones. Overall, from a general perspective, conditions with inflamed samples were again separated from those untreated or starved after inflammation.

#### 3.1.4. Genes Affected by Inflammation and Whose Modulation Is Abolished by Sequential Starvation

**Figure 3:**
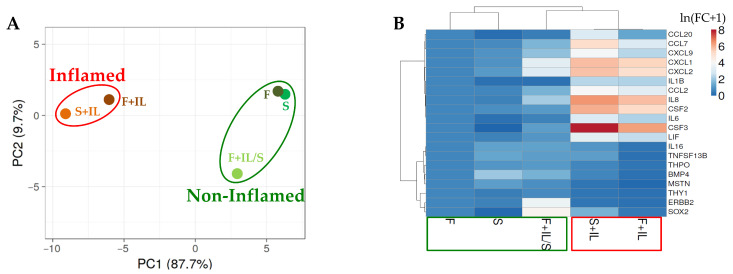
Correlation between samples and conditions for significantly modulated genes: (**A**) PCA performed on ln(FC + 1) values, with FC calculated vs. condition F. The X and Y axes show principal component 1 and principal component 2, which explain 87.7% and 9.7% of the total variance. (**B**) Hierarchical clustering performed on ln(FC + 1) values, with FC calculated vs. condition F.

Eventually, single-gene expression modulation between conditions was analyzed with a specific focus on those genes significantly (*p*-value ≤ 0.05) altered in their amount (calculated as 2^−ΔCt^ with respect to the reference gene) by IL1β (F+IL vs. F or S+IL vs. S) or by starvation after inflammation (F+IL/S vs. F+IL), coupled with up- or downregulation between comparisons higher than five-fold. Twenty players were identified, namely chemokines (*CCL2/7/20* and *CXCL1/2/9*), six growth factors (*BMP4*, *CSF2/3*, *LIF*, *MSTN*, and *THPO*), four interleukins (*IL1B/6/8/16*), two MSC-specific markers (*ERBB2* and *THY1*), a stemness marker (*SOX2*), and a TNF receptor superfamily member (*TNFSF13B*). Computing fold change (FC) values with respect to the F condition, PCA (Figure 3A) again showed that starvation after inflammation (F+IL/S) was able to partially revert the effects of IL1β (F+IL), which were even stronger in the absence of FBS (S+IL) as observed for the analysis of secreted factors in Figure 2. To gain further insights into a single gene’s behavior, hierarchical clustering was performed (Figure 3B). The heat map again emphasized the intermediate position of the F+IL/S condition, with genes clustering under two separate nodes. The first group was rather homogeneous in the gene modulation pattern and composed of *CCL7/20*, *CXCL1/2/9*, *IL1B/6/8*, *CCL2*, *CSF2/3*, and *LIF*. These genes showed strong upregulation under inflammation (F+IL and S+IL) and a partial or total reduction in their amount when starvation was subsequently applied (F+IL/S). The most upregulated players were *CSF3* (vs. F: 535 ± 234-fold upregulation for F+IL, 2693 ± 1648 for S+IL and 2.2 ± 1.4 for F+IL/S; mean ± SEM, N = 3), *IL8* (322 ± 130 for F+IL, 601 ± 182 for S+IL, and 7 ± 1 for F+IL/S), *CSF2* (184 ± 48 for F+IL, 422 ± 185 for S+IL, and 1.9 ± 0.4 for F+IL/S), *CXCL1* (214 ± 105 for F+IL, 336 ± 163 for S+IL, and 27 ± 7 for F+IL/S), *CXCL2* (160 ± 68 for F+IL, 306 ± 127 for S+IL, and 23 ± 5 for F+IL/S), and *CCL7* (22 ± 4 for F+IL, 179 ± 44 for S+IL, and 4 ± 1 for S+IL/S), with IL1β in starvation exhibiting the strongest effect. The second group was characterized by reduced amplitudes of gene modulation. As a general rule for this cluster, all genes were characterized by an increase in expression in F+IL/S vs. F+IL. In particular, the two genes having the highest amplitude were *SOX2* (F+IL/S vs. F+IL: 682 ± 487) and *ERBB2* (99 ± 4). The other five players (*MSTN*, *BMP4*, *TNFSF13B*, *IL16*, and *THPO*) had a less pronounced increase (43 ± 28, 12 ± 2, 8 ± 1, 6.4 ± 0.5, and 5 ± 1) along with their general upregulation in the absence of serum (S and/or S+IL).

#### 3.1.5. ELISA Confirmation of Gene Modulation

**Figure 4:**
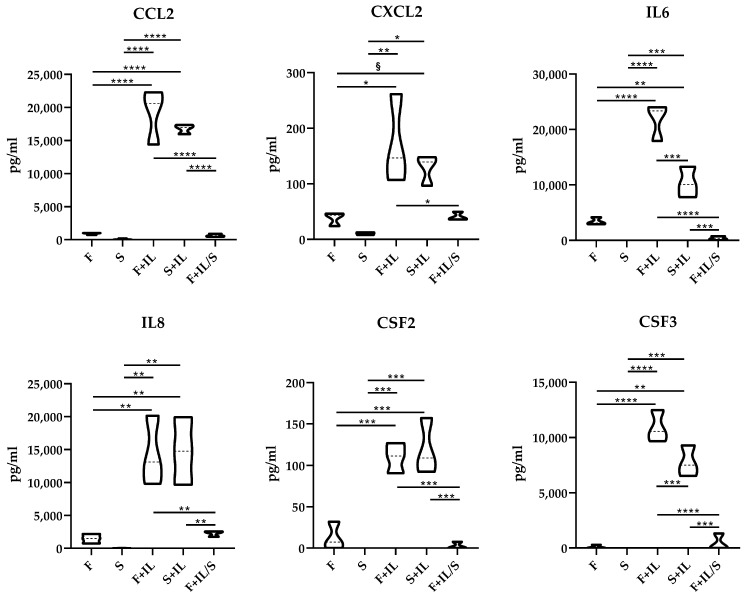
Quantitative analysis of factors modulated by IL1β and reversed by subsequent starvation. CCL2, CXCL2, IL6, IL8, CSF2, and CSF3 levels detected as pg/mL were measured by ELISA assays. In the absence of plots, the proteins were undetectable or below the lower limit of detection of the assay. Under ANOVA analysis, significance was set for *p*-value ≤ 0.05 (§ for *p*-value ≤ 0.1, * ≤ 0.05, ** ≤ 0.01, *** ≤ 0.001 and **** ≤ 0.0001. N = 3).

ELISA assays were performed on the six factors identified in the first cluster of Figure 4 and known to have a role in both MSC function and chondrocyte homeostasis (CCL2, CXCL2, IL6/8, and CSF2/3; Figure 4). Notably, all proteins showed a significant (*p*-value ≤ 0.05) increase in the presence of IL1β (F+IL or S+IL) with respect to their counterparts (F or S). Moreover, starvation after inflammation (F+IL/S) always abolished the upregulation (vs. F+IL). Different from what was observed in gene expression, at the protein level, the factors modulated by IL1β did not reveal an increase in starvation (S+IL), with IL6 and CSF3 being detected at higher levels in F+IL.

#### 3.1.6. Secretomes’ Effect on Chondrocytes

The different secretomes were tested for their anti-inflammatory potential in an in vitro model of chondrocytes treated with IL1β. First, to ensure comparable levels of this cytokine across the different inflamed chondrocyte samples, its amount in the secretomes was tested due to its possible residual presence in the media containing F+IL and S+IL where the molecule was used to prime the ASCs. It revealed assay sensitivity (12 pg/mL) in the F condition, with values of 32 ± 4 pg/mL in S (mean ± SEM, n = 3), 100 ± 19 in F+IL, 108 ± 17 in S+IL, and 27 ± 3 in F+IL/S. Using 1 ng/mL exogenous IL1β to inflame chondrocytes, the total final amount of this cytokine across the treated samples varied, ranging from a 3 to 10% factor between treatments. Also, to potentially discriminate between exogenous IL1β and cell-produced samples in F+IL and S+IL secretomes, a similar approach was used on the stability of IL1β used as input without cells after 48 h at 37 °C. We recovered 36% (365 pg/mL) of the input in the presence of FBS (the base of F+IL secretome) and 16% (162 pg/mL) in the absence of serum (the base of S+IL secretome). The higher values with respect to those measured in the presence of cells suggested a fast turnover and degradation of the exogenous cytokine, with the total values measured in F+IL and S+IL only partially affected by the input remnant. Thus, the exogenous IL1β levels used to inflame chondrocytes were highly comparable across samples, and the final 3–10% difference was mainly dependent on the cell-secreted cytokine.

**Figure 5:**
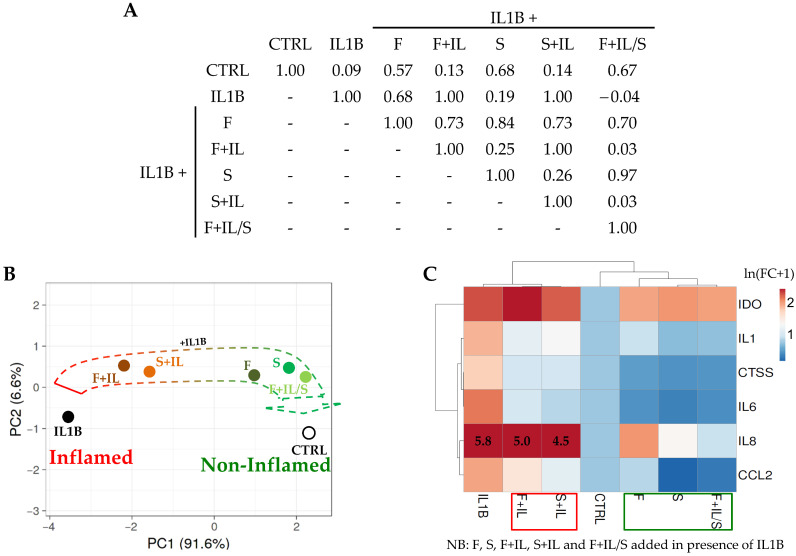
Correlation between conditions for significantly modulated genes in inflamed chondrocytes treated with secretomes: (**A**) Inter-group correlation analysis of the modulation of OA-related genes in chondrocytes treated with IL1β alone or with IL1β and secretomes with respect to untreated (CTRL) cells. “*r*” Pearson values are shown. (**B**) PCA performed on ln(FC + 1) values, with FC calculated vs. condition CTRL. The X and Y axes show principal component 1 and principal component 2, which explain 91.6% and 6.6% of the total variance, respectively. (**C**) Hierarchical clustering performed on ln(FC + 1) values, with FC calculated vs. condition CTRL. The scale bar’s maximum for ln(FC + 1) values was set to 2.5.

The gene expression modulation was tested on six OA and cartilage-damage-related genes, coding for inflammatory cytokines (*IL1/6/8*) and chemokine (*CCL2*), an ECM-degrading peptidase (Cathepsin S, *CTSS*), and a metabolic enzyme inhibiting cartilage regeneration (indoleamine 2,3 dioxygenase 1, *IDO1*). First, correlation analysis between treatments was performed by taking into account the gene modulation values (Figure 5A). As expected, a low *r* value (0.09) emerged for the control (CTRL) vs. inflamed chondrocytes (IL1β). The addition of F+IL and S+IL secretomes to inflamed cells did not reveal an increase in correlation with respect to CTRL, at 0.13 and 0.14, respectively. Notably, F, S, and F+IL/S led to a sharp increase in *r* (0.57, 0.68, and 0.67, respectively), suggesting a reduction in inflammation. Moreover, in the presence of IL1β, F+IL vs. S+IL had an *r* value of 1.00, as was the case with both vs. IL1β alone. As further corroboration of the positive and similar effects of F, S, and F+IL/S, their crossed *r* correlation values were always >0.70, with a higher similarity for S vs. F+IL/S (0.97). Further, F+IL vs. F+IL/S had a very low correlation index (0.03), again suggesting how starvation after inflammation abolished priming effects not only on secreted factors but also on their activity. The overall results were confirmed by PCA analysis (Figure 5B) where inflamed chondrocytes treated with F, S, and F+IL/S were closer to CTRL. Again, F+IL/S and S had the most similar outcome, while F+IL and F+IL/S had the most divergent. Eventually, taking into account single-gene modulation rather than their overall signature, the results of hierarchical clustering and heat map (Figure 5C) clearly demonstrated how secretomes were able to reduce inflammation. Regarding the mean reduction ratios with respect to IL1β, F+IL and S+IL had values of 0.5 ± 0.2 (*p*-value of 0.0374) and 0.4 ± 0.1 (*p*-value of 0.0020), while F, S, and F+IL/S had all values of 0.2 ± 0.1 (*p*-value of 0.0003, 0.0004, and 0.0004, respectively) (mean ± SEM, *n* = 6 genes).

## 4. Discussion

In this work, the modulation of ASCs’ molecular fingerprint was tested during inflammatory priming and subsequent starvation and validated in an in vitro model of OA chondrocytes. The main finding is that secretomes released in the presence of IL1β have a higher number of inflammatory mediators and reduced anti-inflammatory activity on chondrocytes and that starvation after priming abolishes the modulation prompted by inflammation restoring the full potential of ASCs’ secretomes.

The therapeutic features of mesenchymal stromal cells (MSCs) rely on the release of a wide array of soluble factors, known collectively as the “secretome” [15], which is now studied as an innovative cell-free option [16]. A striking example is given by the role of MSCs and the secretome’s potential in musculoskeletal disorders, mainly osteoarthritis (OA), which is explored in both preclinical studies [17,18] and clinical trials [16,19]. Cell culture media or growth conditions have an impact on the potential role of cells and their secreted factors. As an example, the inflammatory environment is pivotal in shaping MSCs’ [20] regulatory role, with inflammation able to polarize MSCs’ secretory portfolio [20,21]. Also, to obtain a clinical-grade secretome, the most used strategy is to remove serum or supplements and apply starvation before conditioned-medium collection [9]. This last step is an underestimated issue potentially affecting cells and released factors’ fingerprints. In this regard, a previous study demonstrated the effect of serum removal on the mitochondrial metabolism of adipose MSCs (ASCs) [10], a crucial regulator of immunomodulation [22]. Therefore, priming strategies able to modulate cells such as inflammation might be potentially watered down by using starvation.

The results of our study clearly demonstrated that in our model with IL1β, starvation after inflammatory priming greatly reduced the molecular signature modulation and preserved the potential of non-inflamed ASCs ‘secretome on chondrocytes in an in vitro 2D model. Under IL1β treatment, regardless of the presence (F+IL) or absence of serum (S+IL), 12 genes were strongly (>5 fold) upregulated (upper cluster in Figure 3B), with ELISA confirming protein increase for 6 players (Figure 4). The majority of the identified factors are known to be involved in immunity at different levels including trafficking (CCL2/7 for monocytes, CCL20 for B and DC cells, CXCL1/2 for neutrophils, and CXCL9 for Th1, B, and NK cells), response (CCL20 for Th17 and CXCL9 for Th1), inflammation (IL1/6/8) and the survival and proliferation of neutrophils and macrophages (CFS2/3) [23]. These results fairly corroborate the literature-reported IL1β inflammation-improved chemoattractant properties of MSCs’ secretome on immune cells [20]. It should be noted that these data were obtained with secretomes collected in the presence of serum. After its removal (F+IL/S), when mimicking GMP procedures to obtain a clinical-grade secretome, we observed a drastic reduction in gene expression upregulation, with protein levels not different from the values in the absence of inflammation (F and S). This result may be due to the combination of inflammatory priming removal and starvation. This molecular signature could explain the differential effects of the secretomes in the in vitro model of OA chondrocytes on factors that have detrimental effects on cartilage and chondrocytes and are involved in OA pathology. CCL2 [24] and CCL20 [25] were shown to be proinflammatory and elicit matrix degradative responses in cartilage, while CCL7 was found upregulated in the synovial fluid of patients with OA [26]. The release of CXCL1 [27] is associated with early cartilage destruction and its increased levels, together with CXCL2 and CSF3, were found to be elevated in arthritic mice [28] as well as CSF2 [29]. A similar trend of upregulation and involvement in OA and cartilage destruction was demonstrated for IL1/6/8 [30], proinflammatory cytokines produced predominantly by activated macrophages and involved in the upregulation of inflammatory reactions [31]. Eventually, LIF [32] is also overexpressed in arthritis and suppresses proteoglycan synthesis [33]. The only protective factor among those elicited by IL1β in ASCs is CXCL9, whose terminal peptide was studied as a therapeutic agent to ameliorate joint inflammation in a murine model of arthritis [34]. Taken together, these lines of evidence explain how secretomes released by ASCs during inflammatory priming (F+IL and S+IL) have a reduced protective activity on OA chondrocytes with respect to those released in the absence of inflammation (F and S), even if previously treated with IL1β (F+IL/S). The overall protective effects of ASCs [35] and their secretomes in the presence [36] and absence [37] of serum consolidate previous reports. Therefore, in view of clinical translation where serum must be avoided, the only available option of secretomes obtained in starvation thus far might be an optimal compromise for cartilage-related disorders balancing a reduced effect on inflammation management and an enhanced activity on chondrocytes’ homeostasis. Also, the concept of ASC priming before secretome collection without serum needs further in-depth analysis since cells under starvation (S) alone can achieve, at least for fingerprint and in vitro effect on chondrocytes, results similar to those observed for F+IL/S.

This work has some limitations. First, the panel of assayed genes was reduced and mainly related to secretory factors and stemness markers. Second, the number of ASC donors was reduced, although with high correlation values. Third, the secretomes’ effect was tested on an in vitro model of immortalized chondrocytes, which only partially reflects what happens in cartilage tissues from OA patients. The presented results are meant as a first screening to identify on a molecular level and in a simple in vitro test the most effective condition for cartilage-related applications. Future works will be directed toward confirming these results in more complex 3D models, such as chondrogenic pellets and microfluidic approaches using OA patients’ chondrocytes or ex vivo OA cartilage samples that more closely resemble the in vivo environment and response to pathological stimuli. Eventually, we opted for starvation after inflammation to mimic what happens during the clinical production of secretome while being aware that adding a complete medium after IL1β priming could lead to different results; this needs to be further investigated.

## 5. Conclusions

The results reported in this manuscript highlight how production processes might influence MSCs’ fingerprint and released factors envisioned as next-generation therapeutic products. Actual strategies to obtain serum-free secretomes might modulate the properties of primed MSCs, prompting an in-depth analysis of these new approaches for the use of released factors in translational medicine.

## Data Availability

Raw data are available at: https://osf.io/eadps/?view_only=9637dfc380ee450fbfdab1c4cd3d641e (accessed on 22 March 2024).
